# Temporal Trends in Spinal Imaging in Ontario (2002-2019) and Manitoba (2001-2011), Canada

**DOI:** 10.7759/cureus.63267

**Published:** 2024-06-27

**Authors:** Rayeh K Al-Ghetaa, Mostafa Alabousi, John J You, Peter C Emary, John J Riva, John Dufton, Yoan K Kagoma, Raja Rampersaud, Michael J Goytan, Thomas E Feasby, Martin Reed, Jason W Busse

**Affiliations:** 1 Institute of Health Policy, Management and Evaluation, University of Toronto, Toronto, CAN; 2 Radiology, McMaster University, Hamilton, CAN; 3 Medicine, Trillium Health Partners, Mississauga, CAN; 4 Anesthesia, McMaster University, Hamilton, CAN; 5 Family Medicine, McMaster University, Hamilton, CAN; 6 Imaging, University Hospital of Northern British Columbia, Prince George, CAN; 7 Medical Imaging, McMaster University, Hamilton, CAN; 8 Surgery, University of Toronto, Toronto, CAN; 9 Surgery, University of Manitoba, Winnipeg, CAN; 10 Clinical Neurosciences, University of Calgary, Calgary, CAN; 11 Radiology, University of Manitoba, Winnipeg, CAN; 12 Health Research Methodology, McMaster University, Hamilton, CAN

**Keywords:** magnetic resonance imaging, computed tomography, x-ray, cost, imaging, spine

## Abstract

Background

Several studies have reported the overuse of spinal imaging, which, in Canada, led to several provincial pathways aimed at optimizing the use of imaging. We assessed temporal trends in spine imaging in two Canadian provinces.

Methods

We explored the use of X-ray, computed tomography (CT), and magnetic resonance imaging (MRI) examinations of the cervical, thoracic, and lumbar spine regions among adults in Ontario (April 1, 2002, to March 31, 2019) and in Manitoba, Canada (April 1, 2001, to March 31, 2011) using linked Ontario Health Insurance Plan administrative databases and data from Manitoba Health. We calculated the age- and sex-adjusted rates of spinal X-ray, CT, and MRI examinations by dividing the number of imaging studies by the population of each province for each year and estimated the use of each imaging modality per 100,000 persons.

Results

The total cost of spine imaging in Ontario increased from $45.8 million in 2002/03 to $70.3 million in 2018/19 (a 54% increase), and in Manitoba from $2.2 million in 2001/02 to $5 million in 2010/11 (a 127% increase). In Ontario, rates of spine X-rays decreased by 12% and spine CT scans decreased by 28% over this time period, while in Manitoba, rates of spine X-rays and CT scans remained constant. Age- and sex-adjusted utilization of spinal MRI scans per 100,000 persons markedly increased over time in both Ontario (277%) and Manitoba (350%).

Conclusion

Despite efforts to reduce the use of inappropriate spinal imaging, both Ontario and Manitoba have greatly increased utilization of spine MRI in the past two decades.

## Introduction

Approximately 80% of adults will experience spine-related complaints (e.g., low back, mid-back, or neck pain) in their lifetime, and lumbar spine imaging accounts for about one-third of all magnetic resonance imaging (MRI) in many jurisdictions [1]. In Canada, between 1993 and 2004, the number of computed tomography (CT) scans grew by 300% and the number of MRI scans grew by 600% [2]. Contributing factors to the increased use of imaging include increased physician reliance on technology, financial incentives, patient demand, spine surgeons’ requirements for imaging prior to consultation, and defensive medicine [3-7]; however, with the exception of ‘red flag’ diagnoses (e.g., spinal neoplasms, fractures, infections, inflammatory arthritis, cauda equina syndrome), clinical practice guidelines have concluded that imaging for patients with uncomplicated spine complaints does not improve outcomes [8-12].

A 2011 study of patients with degenerative spine disease referred for surgical consultation in Ontario, Canada found that 100% of CT scans and 60% of MRIs were unnecessary, associated with an additional cost of 24 million dollars/year [13]. Another study found that over half of lumbar spine MRIs in Edmonton and Ottawa, Canada, ordered between 2008 and 2010, were either inappropriate or of uncertain value [14]. Accordingly, Choosing Wisely Canada, which was launched in April 2014, has recommended against diagnostic imaging for uncomplicated low back or neck pain [15,16], and care pathways have been developed in several Canadian provinces to improve imaging appropriateness [17]. We explored the utilization and associated costs of X-ray, CT, and MRI for cervical, thoracic, and lumbar spine-related complaints in Ontario and Manitoba over the prior two decades.

## Materials and methods

Study design and data sources

All imaging studies ordered by medical professionals in Ontario and Manitoba, Canada, are funded by provincial health plans, the Ontario Health Insurance Plan (OHIP), and Manitoba Health. We conducted a retrospective study using linked provincial health administrative databases to examine spine imaging utilization in Ontario from April 1, 2002, to March 31, 2019, and Manitoba Health data from April 1, 2001, to March 31, 2011. We followed the REporting of studies Conducted using Observational Routinely-collected health Data (RECORD) statement [18].

The OHIP claims database is based on fee-for-service reimbursement claims made by physicians to the Ministry of Health and Long-Term Care (MOHLTC), using fee codes listed in Table 1. For X-ray imaging, fee codes allow the stratification of imaging regions of the spine into cervical, thoracic, and lumbar; however, for CT and MRI, the OHIP fee codes are the same for all regions of the spine. Manitoba Health collects and records all physician claims data, including spinal imaging procedures, and the fees paid for these procedures.

**Table 1:** Eligible Ontario Health Insurance Plan (OHIP) fee codes

Description	OHIP fee code
Spine X-ray	X025, X027, X028, X031, X032, X033, X034, X035, X128, X202, X203, X204, X205, X206, X207, X208
CT spine	X128, X415, X416
MRI spine	X490, X492, X496

Demographic information

We accessed the Ontario Registered Persons Database (RPDB) to acquire the age and sex of all patients who underwent spine imaging in Ontario. Both the MOHLTC and RPDB administrative datasets are housed at the Institute for Clinical Evaluative Sciences (ICES). ICES is an independent, non-profit research institute whose legal status under Ontario’s health information privacy law allows it to collect and analyze health care and demographic data, without consent, for health system evaluation and improvement. Median income data for patients living within a certain geographic region was derived based on postal codes obtained from the Statistics Canada Postal Code Conversion File. Similar information on demographics was available through Manitoba Health. Annual population estimates were obtained from the Statistics Canada census. We used national census data to obtain data regarding patients’ place of residence at the time a given spinal imaging procedure was performed to stratify “urban” versus “rural” populations and to stratify by neighborhood income quintile. Previous studies have shown that diagnostic imaging utilization is correlated with geographic region and socioeconomic status [19,20]. We categorized population density as rural if the total population was <10,000 [21].

Statistical analysis

We calculated the number of each type of spinal imaging procedure performed in each calendar year in Ontario (between April 1, 2002, and March 31, 2019) and in Manitoba (between April 1, 2001, and March 31, 2011). The annual period was defined based on the fiscal year, beginning on April 1 and ending on March 31 of the following year. For calculation of the rates of annual spine imaging, the numerator was defined as the total number of corresponding OHIP or Manitoba Health fee code entries for the specific imaging modality during the predefined annual range. The denominator was the annual Ontario or Manitoba population estimate for adults (aged 18 years) according to the Statistics Canada census. Frequencies were obtained, as well as age- and sex- standardized rates per 100,000 people. Patient income was categorized into neighborhood income quintiles (NIQ).

The annual costs of each spinal imaging modality were calculated by multiplying the number of examinations performed for the modality (according to its associated fee code) by the corresponding billing fee published within the OHIP schedule of benefits or Manitoba Health fee schedules [22]. In Ontario, the total of the professional and technical fees for X-ray imaging was reported for the cost, whereas only professional fees were included for CT and MRI since the Ontario model relies on hospitals absorbing the technical fees for CT into their global operating budget while MRI technical fees are funded separately by the MOHLTC. For the Manitoba cost data, only professional physician fees could be obtained and included in the overall calculations. 

We performed all analyses using Stata V.16.1 (StataCorp, College Station, TX, USA).

Ethics approval

Our project received an exemption waiver from the Hamilton Integrated Research Ethics Board (HiREB) at McMaster University based on the data being solely obtained and analyzed by ICES.

## Results

Ontario

Overall Imaging Rates

**Figure 1:**
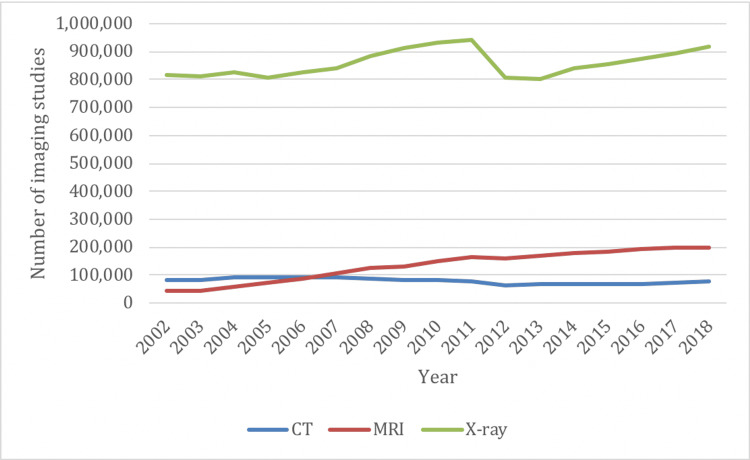
Overall spine imaging rates in Ontario by imaging modality from 2002/03 to 2018/19

In 2002/03, Ontario reported a total of 941,952 spinal imaging examinations, of those 816,544 (87%) were X-rays, 83,468 (9%) were CTs, and 41,940 (4%) were MRIs. In 2018/19, the province reported a total of 1,194,499 spinal imaging examinations, of those 917,318 (77%) were X-rays, 79,626 (7%) were CTs and 197,555 (16%) were MRIs. The change from 2002/03 to 2018/19 represents an increase of 27% for spine imaging, including a 12% increase for X-rays, a 5% decrease for CTs, and a 371% increase for MRIs (Figure 1).

**Figure 2:**
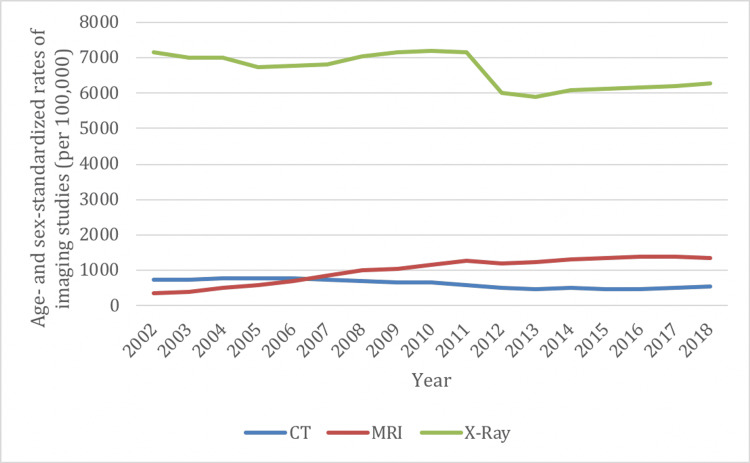
Age- and sex-standardized spine imaging rates in Ontario by imaging modality from 2002/03 to 2018/19

Age- and sex-standardized rates of spinal imaging examinations decreased between 2002/03 and 2018/19 for X-ray (7,171 vs. 6,282 per 100,000 persons) and CT (735 vs. 530 per 100,000 persons) while the rates increased for MRI (363 vs. 1,367 per 100,000 persons) (Figure 2). The change from 2002/03 to 2018/19 represents an overall decrease in spinal imaging of 1% (12% decrease for X-rays, 28% decrease for CTs); however, there was a 277% increase for MRIs.

Imaging Rates by Age Group

**Figure 3:**
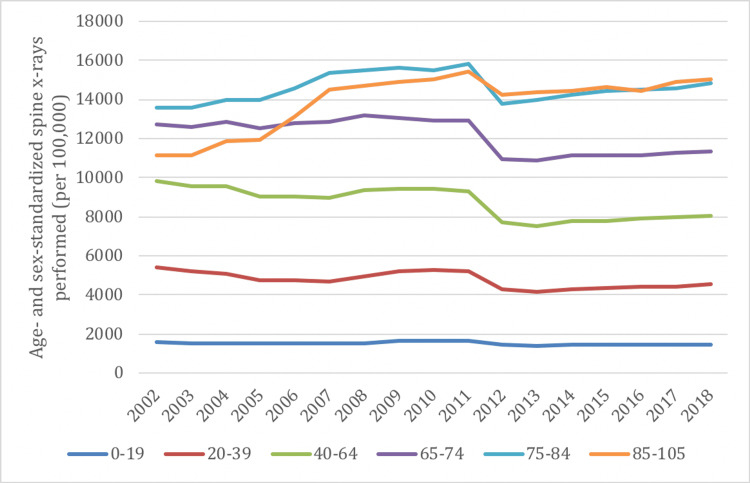
Age- and sex-standardized spine imaging rates in Ontario stratified by age group for X-ray from 2002/03 to 2018/19

Similar to the trends in overall imaging rates, there was a decrease in the rate of spine X-rays between 2002 and 2019 in all age groups, ranging from decreases of 10% to 18%, except for older adults, which demonstrated increases of 9% for those age 75-84, and 35% for those age 85-105 (Figure 3). Spine X-rays were most frequently performed in those ≥75 years of age, and least frequently performed in the ≤19 age group.

**Figure 4:**
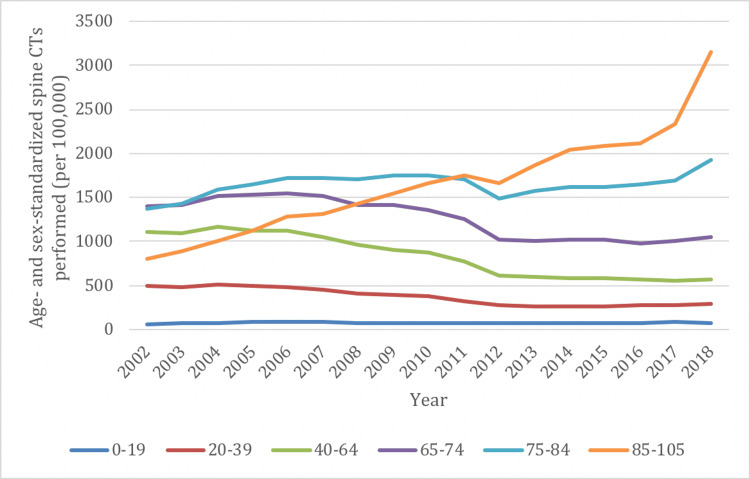
Age- and sex-standardized spine imaging rates in Ontario stratified by age group for CT from 2002/03 to 2018/19

Spine CTs in the 20-39, 40-64, and 65-74 age groups demonstrated decreased rates of utilization between 2002/03 and 2018/19, ranging from decreases of 25% to 48% (Figure 4). However, the 75-84 age group demonstrated a moderate increase (41%) and the 85-105 age group demonstrated a marked increase (290%) in spine CT utilization. The ≤19 age group demonstrated a low overall rate of spine CT, with a modest increase (19%) over time. Similar to X-ray utilization, those ≥75 years of age had the highest overall rates of spine CT while those aged ≤19 years had the lowest utilization. 

**Figure 5:**
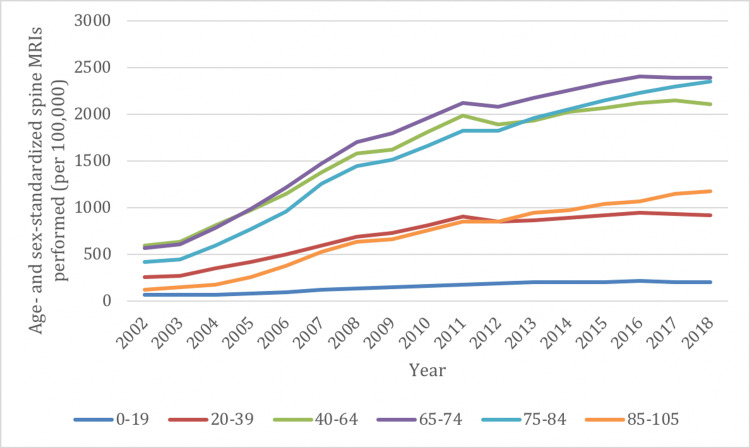
Age- and sex-standardized spine imaging rates in Ontario stratified by age group for MRI from 2002/03 to 2018/19

Spine MRIs increased between 2002/03 and 2018/19 in all age groups, with the most pronounced increase in utilization seen in the 85-105 (855%) and 75-84 (468%) age groups (Figure 5). The rate of increase in the remaining age groups ranged from 230% to 323%. The highest utilization of spine MRI was seen in the 65-74 age group while the lowest utilization was in those aged ≤19 years.

Imaging Rates by Neighborhood Income Group

**Figure 6:**
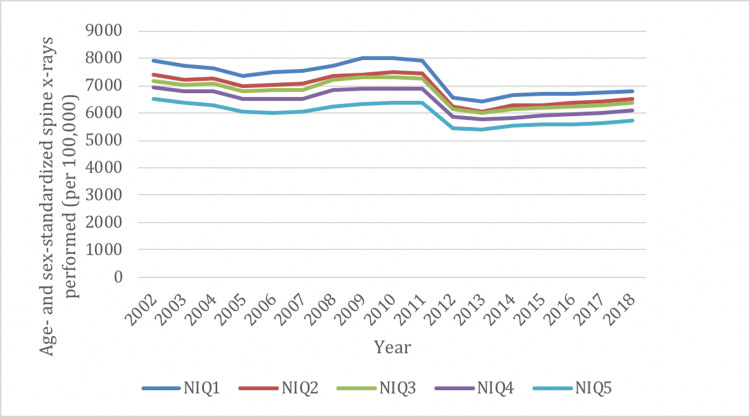
Age- and sex-standardized spine imaging rates in Ontario stratified by neighborhood income quintile (NIQ) for X-rays from 2002/03 to 2018/19

**Figure 7:**
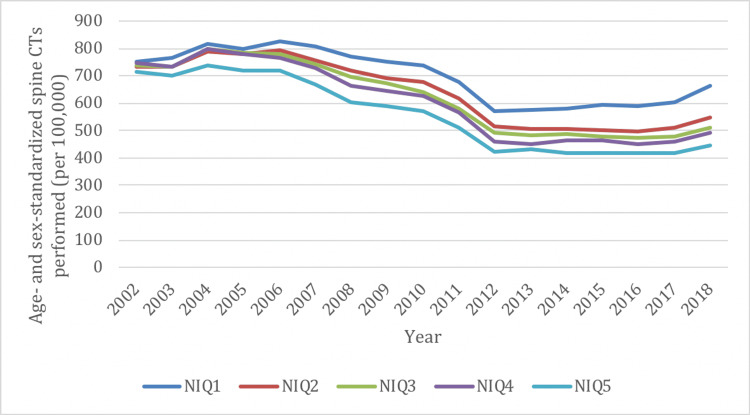
Age- and sex-standardized spine imaging rates in Ontario stratified by neighborhood income quintile (NIQ) for CT from 2002/03 to 2018/19

**Figure 8:**
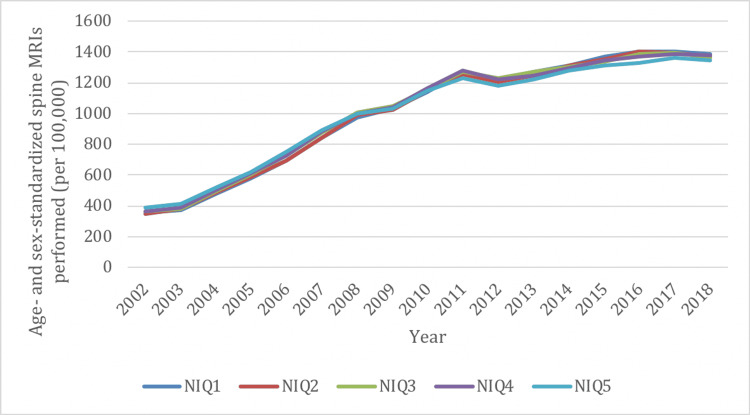
Age- and sex-standardized spine imaging rates in Ontario stratified by neighborhood income quintile (NIQ) for MRI from 2002/03 to 2018/19

Similar to overall rates, there was a decrease in spine X-ray utilization between 2002 and 2019 in all NIQ groups (range, -11% to -14%) (Figure 6). Spine X-rays were most frequently performed in the highest neighborhood income group (NIQ1) and least frequently performed in the lowest neighborhood income group (NIQ5). Spine CTs also demonstrated a decreased rate of utilization between 2002 and 2019 in all NIQ groups (range, -12% to -38%) (Figure 7), and were most frequently performed in the NIQ1 group and least frequently performed in the NIQ5 group. There was a marked increase in the rate of spine MRIs between 2002 and 2019 in all NIQ groups (range, 247% to 294%), and this was comparable across all income quintiles (Figure 8).

*Costs* 

**Figure 9:**
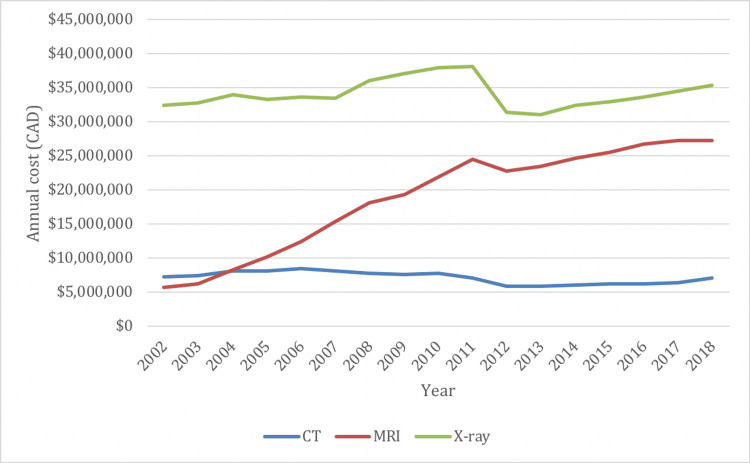
Overall cost of spine imaging in Ontario by imaging modality from 2002/03 to 2018/19

In 2002/03, Ontario reported a total cost of $45.8 million for spine imaging examinations: $32.4 million for X-rays, $7.3 million for CTs, and $5.6 million for MRIs. The total cost of spine imaging rose to $70.3 million by 2018/19: $35.3 million for X-rays, $7.0 million for CTs, and $27.2 million for MRIs. The change in total cost between 2002/03 and 2018/19 represented an increase of 54%: a 9% increase for X-rays, a 4% decrease for CTs, and a 386% increase for MRIs (Figure 9).

Manitoba

**Figure 10:**
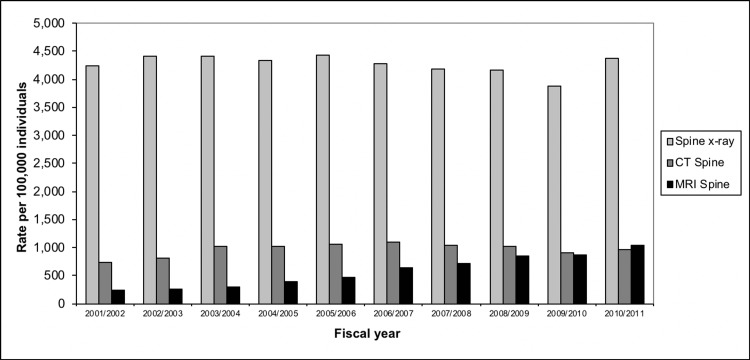
Spine imaging rates (age-, sex-adjusted), Manitoba, 2001-2011

The overall age- and sex-adjusted population rates of spine X-ray, CT, and MRI are shown in Figure 10, reported as rates per 100,000. The rates of spine X-ray and CT imaging were relatively stable between 2001 and 2011. However, the rate of spine MRIs increased by 4.5 times (350%) during this time period.

Imaging Rates by Neighborhood Income Group

**Figure 11:**
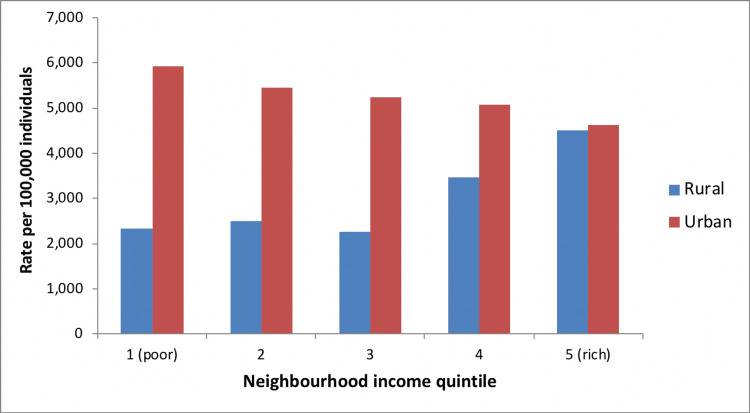
Spine X-ray use by neighborhood income, Manitoba (2010/11)

**Figure 12:**
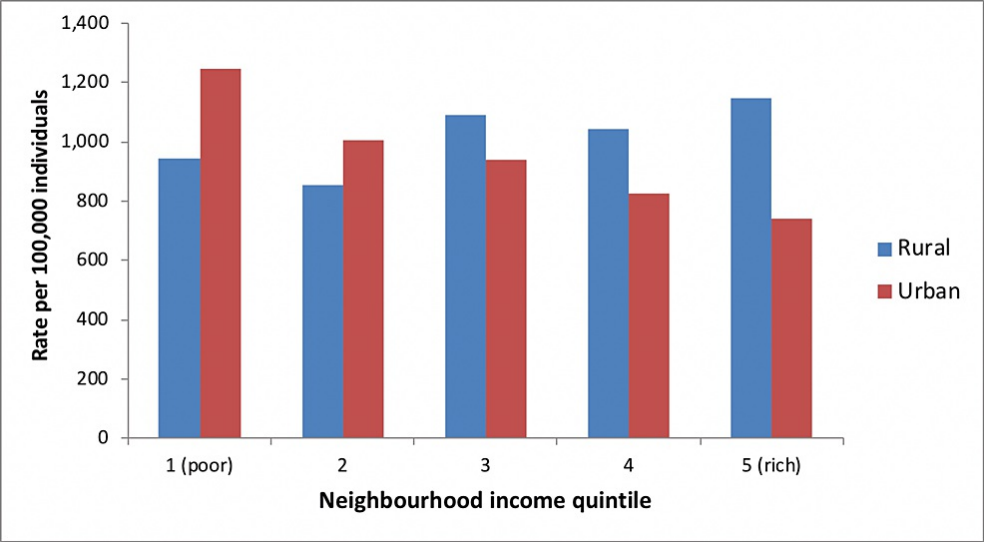
CT spine use by neighborhood income, Manitoba (2010/11)

**Figure 13:**
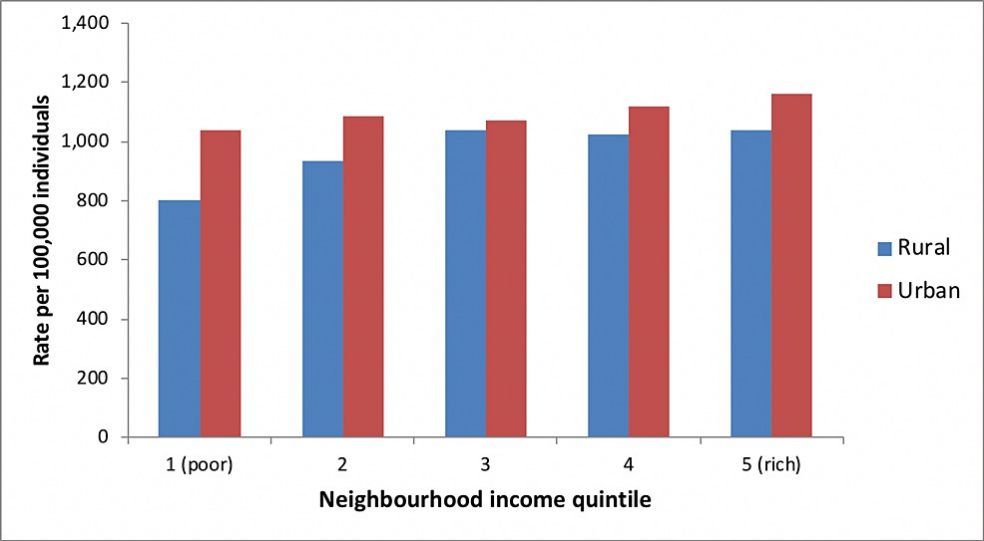
MRI spine use by neighborhood income, Manitoba (2010/11)

In urban areas, higher neighborhood income was associated with higher rates of spine MRI utilization, but lower rates of spine X-ray or CT utilization. In rural areas, utilization of spinal imaging was greater (regardless of imaging modality) among individuals with higher socioeconomic status (Figures 11-13). The total cost of spinal imaging (X-ray, CT, MRI) was $2.2 million in 2001/02. This increased to $5 million in 2010/11, an increase of 127% over the 10-year period. Although spending increases were greatest for spine MRI, costs of spine X-ray were $2 million in 2010/11, which accounted for 40% of total spine imaging costs in Manitoba and was equal to expenditures on spinal MRI.

## Discussion

Our study investigated the rates of all spinal X-ray, CT, and MRI examinations among adults over the previous two decades in Ontario (2002 to 2019) and Manitoba (2001 to 2011), Canada. We found a 27% increase in the rate of spine imaging utilization in Ontario, mainly driven by a greater than four-fold increase in the rate of MR spinal imaging. Over 18.2 million spinal X-ray, CT, and MR imaging examinations were performed in Ontario between 2002 and 2019, costing the province over $1 billion (CAD). Costs attributable to spinal imaging rose by 54% during this time, driven primarily by increased use of MRI. Similar trends were seen in Manitoba, where the rate of spine MRI utilization increased 4.5-fold and constituted 40% of total spine imaging costs provincewide as of 2010/11.

In contrast with MRI, we found that overall rates of spine X-ray and CT imaging declined or remained stable in Ontario and Manitoba; however, Ontarians over the age of 75 demonstrated increasing rates of X-ray and CT utilization over the 2002 to 2019 study period. Reduced bone mineral density with increasing age, as well as a higher risk of accidental falls, raises the fracture risk in elderly patients and may explain the higher rate of first-line X-ray imaging amongst older patients [23,24]. We found a pronounced reduction in the utilization of spinal X-rays and CTs in Ontario in 2012, and this may be related to an amendment to the Schedule of Benefits for Physician Services [25] that limited the eligibility of lumbar spine imaging payment to cases of low back pain with suspected or known pathology [26].

Our observation of increased MRI utilization compared with relatively steady or decreasing rates of spine X-rays and CT imaging over time in Ontario and Manitoba likely reflects MRI’s replacement of CT as the “gold standard” imaging modality for spine-related complaints such as spinal cord and nerve root pathologies [27]. Results of a 2014 survey of Canadian spine surgeons [7] found that 78% of respondents required imaging studies to accompany all physician spine-related referrals, most commonly MRI (48%), possibly further contributing to this trend. Of note, a prior study has found that most MRIs requested for surgical referrals are unnecessary [28]. An awareness among physicians and patients of the risks associated with ionizing radiation exposure of X-rays and CT imaging may have also played a role in the trends we observed [27].

The rising use of imaging for spine-related complaints is incongruent with prior [29] and current imaging guidelines and recommendations [8-12,15,16], many of which were already in place at some point during the 2002/19 (Ontario) and 2001/11 (Manitoba) time periods investigated. The prevalence of inappropriate spine imaging has been well-documented, with previous reports indicating guideline-discordant imaging in up to 54% of cases involving the cervical spine and 80% for the lumbar spine [1]. A prospective analysis of outpatient MRI requisitions at two Canadian hospitals [14] found that in 56% of patients referred for lumbar spine MRI, the imaging study was deemed inappropriate or of uncertain value. Similarly, a 2018 systematic review and meta-analysis of 33 studies concluded that lumbar spine X-ray, CT or MR imaging was inappropriately performed 44% of the time in patients presenting for care, as judged by duration of episode (28%; 95% CI, 21-35%), absence of red flags (9%; 95% CI, 7-11%), or lack of clinical suspicion of pathology (7%; 95% CI, 2-23%) [10]. Previous research has also demonstrated a poor correlation between lumbar spine diagnostic imaging findings, clinical symptoms [30], and healthcare utilization [31]. Patients’ expectations and physicians’ fear of litigation have been identified as common reasons for inappropriate spine imaging referrals [14].

Excessive or inappropriate utilization of diagnostic imaging for spine-related complaints in hospital-based and primary care settings may be related to poor implementation of accepted criteria for appropriate spine imaging use [2,32]. For example, acquiescing to a patient’s request for imaging is more efficient than explaining why a given test is either of uncertain value or inappropriate. Our results and those of other studies [13,14,28] suggest that ongoing overutilization of spine-related imaging in Canada and elsewhere [10] poses a significant burden on healthcare systems, and limits timely access to diagnostic imaging for patients most in need [14,33]. Accordingly, there is a need for implementation and enforcement of clinical decision supports [34] and spine care pathways, such as the Rapid Access Clinics for Low Back Pain program in Ontario, Canada [35], where a 31% reduction in MRI referrals was demonstrated within the first three years of its inception [17]. Unnecessary imaging also poses risks to patients, including false positive tests leading to psychological stress and unnecessary invasive procedures, as well as the risk with X-rays and CT scans of exposure to ionizing radiation [28,32]. Understanding current utilization trends may help guide future health system interventions aimed at improving the appropriateness of spine imaging [1,17].

Limitations

This study had several limitations. Due to the lack of clinical findings and outcomes, we were unable to characterize rates of appropriate imaging use; however, several prior studies [13,14,28] have found that most spinal MRIs are unnecessary. Second, our cost analyses of imaging are underestimated as technical fees/operating budgets could not be included. Further, most data available for our study was from ICES, which only provides healthcare utilization for Ontario and may limit the applicability of results in other jurisdictions. We included data from Manitoba, available to the year 2010/11, to increase the generalizability of our findings. We relied on historical data and were unable to examine imaging rates or appropriateness in different clinical settings (e.g., outpatient vs. emergency department) or between different referring physician groups (e.g., family doctors, radiologists, nurse practitioners, chiropractors, or physiotherapists) because this information was not available in the databases utilized. The inability of the accessed databases to divide CT and MR spinal imaging into cervical, thoracic, and lumbar levels is also a limitation. We also did not control for additional confounding factors (e.g., operating room access), or include a comparison group to determine if the rate of spine MRI increased at a higher rate than other musculoskeletal or organ systems (e.g., knee, liver). These data would provide insight into whether the increased rates of spinal MRI utilization were related to increased MRI capacity (i.e., reduced wait times for appropriate imaging) versus inappropriate spinal imaging, as implied.

## Conclusions

There has been a large increase in the use of costly spine MR imaging between 2002 and 2019 in Ontario, Canada, with a similar trend observed in Manitoba between 2001 and 2011. Enforcement of evidence-based policies is needed to optimize the use of spinal imaging and healthcare investment.
